# Prospective *In Vitro* Models of Channelopathies and Cardiomyopathies

**DOI:** 10.1155/2012/439219

**Published:** 2012-03-27

**Authors:** Nanako Kawaguchi, Emiko Hayama, Yoshiyuki Furutani, Toshio Nakanishi

**Affiliations:** Department of Pediatric Cardiology, Tokyo Women's Medical University, 8-1, Kawada-cho, Shinjuku-ku, Tokyo 162-8666, Japan

## Abstract

An *in vitro* heart disease model is a promising model used for identifying the genes responsible for the disease, evaluating the effects of drugs, and regenerative medicine. We were interested in disease models using a patient-induced pluripotent stem (iPS) cell-derived cardiomyocytes because of their similarity to a patient's tissues. However, as these studies have just begun, we would like to review the literature in this and other related fields and discuss the path for future models of molecular biology that can help to diagnose and cure diseases, and its involvement in regenerative medicine. The heterogeneity of iPS cells and/or differentiated cardiomyocytes has been recognized as a problem. An *in vitro* heart disease model should be evaluated using molecular biological analyses, such as mRNA and micro-RNA expression profiles and proteomic analysis.

## 1. Introduction

Most of the genes responsible for congenital heart diseases have been identified with genetic studies, where healthy individuals and patients' genes sequences were compared to find mutations. The responsible genes were then subjected to functional analyses, using knock-out mouse and/or other animals to make a disease model which possessed the mutated genes [1, 2].

Since their establishment [3–5], iPS cells have been used to make *in vitro* disease models because of the difficulty in using a patient's cells or tissues, especially from the heart [6–9]. Transfection of mutated genes into a normal parent cell prior to formation of iPS cells has also been used to make an *in vitro* disease model. Thus, iPS cells or differentiated cells containing the mutated gene can be compared with parent cells that do not have the mutated gene. ES cells and iPS cells differentiate into heart cells more easily than adult cardiac stem cells in both mice and humans because of their multipotency and pluripotency characteristics. Therefore, these cells have been used in regenerative medicine studies [10–12]. Although cardiac stem cells have advantage for *in vivo* regenerative medicine [13, 14], heterogeneity was observed in long-term cultures in our *in vitro* cultures [15]. A previous report showed that immature cardiomyocytes were obtained *in vitro* differentiation [16], suggesting the limitations of using adult stem cells as a cell source for *in vitro* disease model. Taken together, ES cells/iPS cells provide a better cell source of cardiomyocytes required for *in vitro* disease models.

In heart disease, iPS cells from Long-QT-syndrome-(LQTS-) type1 [17] and LQTS-type2 [18] patients were made and differentiated cardiomyocytes were obtained from these iPS cells. These cardiomyocytes worked as *in vitro* heart disease models since they possessed similar characters to patients' cardiomyocytes. LQT1 and LQT2 are caused by missense mutations of the *KCNH1* and *KCNH2* gene, respectively. These mutations in potassium channels lead to QT interval prolongation [19]. Interestingly, the differentiated cardiomyocytes also showed marked arrhythmogenicity and early afterdepolarizations [18]. Potassium channel activators, such as PD118057, cured prolonged action potentials of LQT2-hiPS cell-derived cardiomyocytes [20]. Cardiomyocytes derived from patients' fibroblasts, or other somatic cells, are gaining attention as promising models to discover drug targets for disease.

The differentiated cardiomyocytes from murine iPS cells, mutated with the LQT3 gene (Scn5a^Δ/+^), showed prolonged action potentials because of a Na channel dysfunction mutation in an LQTS-type3 patient [21], suggesting even murine iPS-derived cardiomyocytes can be used for an *in vitro* disease model. iPS cell-derived cardiomyocytes from Timothy syndrome showed irregular contractility consistent with the disease phenotype [22]. At least 13 LQTS genes have been reported so far, and similar abnormalities in iPS-derived cardiomyocytes from patients can be anticipated.

 Channelopathies have been currently used as *in vitro* disease models because of the development of systematic current measurements. Another recent model from channelopathy was catecholaminergic polymorphic ventricular tachycardia (CPVT), carrying a novel mutation (S406L) of the ryanodine receptor (RYR) 2 which reduced sarcoplasmic reticular (SR) Ca^2+^ content to levels lower than control myocytes. In this case, Dantrolene is a drug rescued arrhythmogenic phenotype [23]. LEOPARD (lentigines, electrocardiographic abnormalities, ocular hypertelorism, pulmonary valve stenosis, abnormal genitalia, retardation of growth, and deafness) syndrome is caused by a different missense mutation of the *PTPN11* gene (T468M and Y279C are the most recurrent). Differentiated cardiomyocytes from these patients were larger than wt-iPS-cell- or ES-cell-derived cardiomyocytes, which correspond to the disease phenotype of LEOPARD cardiac hypertrophy [24].

Another attractive method can be direct programming into stem cells/progenitors/cardiomyocytes from patients' somatic cells. iPS cells induced from adult neural stem cells with only one transcription factor (TF), Oct4, were similar to ES cells [25]; therefore, primitive cells may be more suitable than differentiated cells to make iPS cells with only one factor introduction. Transient introduction of Yamanaka 4 factors (Oct3/4, Sox2, Klf4, and c-myc) and immediate growth factors, mainly bone morphogenetic protein 4 (BMP4), to cultured cells adequately directed cardiomyogenesis [26]. Interestingly, direct reprogramming from fibroblasts into cardiomyocytes was successful using 3 TFs, which are associated with cardiomyogenesis [27], which is another possible method of producing cardiomyocytes.

## 2. Generation of iPS Cells from Patients

**Figure 1:**
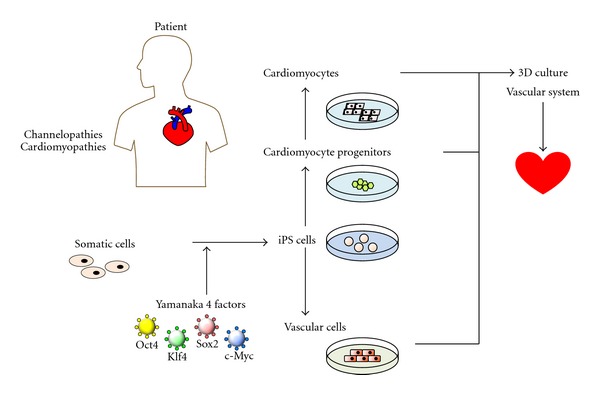
Whole concept of *in vitro* disease model for heart disease (channepathies and cardiomayopathies).

Although a retrovirus was originally used, recently there have been several methods of reprogramming developed to introduce Yamanaka 4 factors (Figure 1). The Sendai-virus [28, 29], transient transfection of mRNA [30, 31], is more attractive than conventional retroviral infections because of safety, which is important for regenerative medicine and also *in vitro* models. If reprogramming vectors are integrated into the host genome, tracking the location can be difficult. Moreover, additional artifacts are also a concern. Recent studies show that epigenetic modulators such as the histone deacetylase inhibitor, valproic acid (VPA) can affect reprogramming efficiency [32]. In this way, only two factors (Sox2 and Oct4) efficiently induce iPS cells [33]. The butyrate [34] DNA methyl transferase inhibitor, RG108 [35], improves the efficiency of skeletal myoblast reprogramming. Interestingly, cardiomyocytes differentiated from these skeletal myoblast-derived iPSs (SiPS) improved the cardiac function of an infarcted heart without tumorgenesis [35, 36]. Epigenetic studies of reprogramming and stemness have attracted the interest of many researchers [37–40]. Indeed, hot spots are investigated that are difficult to methylate [41]. Therefore, more efficiency is expected by identifying and modifying these spots.

Congenital heart diseases, modified from the work of Ackerman et al. [42], are summarized in Table 1. The diseases of channelopathies and cardiomyopathies are listed and summarized with experts evaluation, “is recommended” or “not is recommended,” according to the present characterization of gene mutations. Currently, channelopathies have been well characterized because of systematic measurements of cardiomyocytes or beating embryonic bodies (EBs). These diseases are candidates for *in vitro* models from iPS cells. Recently, iPS cell-derived cardiomyocytes from Pompe disease, known as a glycogen storage disease, were established and were revealed to have higher glycogen contents than hESC and control iPS-derived cardiomyocytes [43]. The generation of iPS-derived cardiomyocytes from these patients is expected to provide important information about these diseases.

## 3. Generation of Cardiomyocytes from iPS Cells

**Figure 2:**
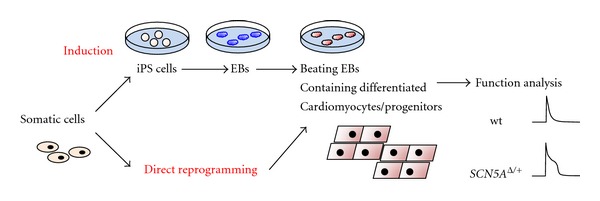
The methodology for *in vitro* cardiomyocyte differentiation.

 The differentiation method from iPS cells into cardiomyocytes basically follows the protocol of embryonic stem (ES) cells, using embryonic bodies (EBs, see Figure 2). Yang et al. showed that KDRlow/C-KIT neg EBs differentiated into cardiomyocyte lineages and became NKX2.5, ISL1, TBX5 positive but not KDRlow/C-KITpos or KDRneg/C-KITpos [44]. The combination of activin A, BMP 4, basic fibroblast growth factor (bFGF), vascular endothelial growth factor (VEGF), and Dickkopf homolog 1 (DKK1) in a serum-free media was necessary for cardiomyogenesis. Likewise, addition of Wnt inhibitors to BMP 4 enhanced cardiomyogenesis [45]. These activin/nodal and BMP signaling pathways promote cardiac differentiation in a stage-specific manner [46]. The role of c-kit may be different even in the embryonic stage, since c-kit high-expressing cells became cardiomyocytes and other cardiac cell lineages near birth [47]. The level and timing of c-kit expression can change its role [48]. Flk-1+ cells from EB clusters are produced in ES cell cultures without LIF, and cardiac progenitors and cardiovascular cells were also formed from these EB clusters [49, 50].

 Cardiomyocytes obtained from iPS cells were functionally similar to ES cells-derived cardiomyocytes [51], and multiple type action potential (nodal, atrial, and ventricular) phenotypes were observed [52]. Overall, the gene expression profiles of iPS cells were similar to ES cells, but differentiation direction and efficiency were variable [53, 54]. Overall, iPS-cell-derived cardiomyocytes have similar contractile behaviors to ES cell-derived cardiomyocytes but are significantly different from native tissues from comparable ages [52]. However, the drug effect on iPS-cell-derived cardiomyocytes is similar to cardiomyocytes derived from hES cells [55]. As a cell source, ventricular cardiomyocytes produced more cardiomyocytes than somatic cells such as tail-tip fibroblasts [56]. The variability of differentiation among the cell lines has been previously reported [57]. The heterogeneity of iPS-derived cardiomyocytes is a problem for establishing good models [58]. One of the solutions is to obtain extremely pure cardiomyocytes to eliminate heterogeneity as much as possible. Ma et al. selected highly purified iPS cell-derived cardiomyocytes using blastcidin resistance gene expression controlled from the cardiac-specific endogenous MYH6 promoter and investigated drug electrophysical properties [59]. Another method used to eliminate heterogeneity was to establish a systematic protocol which produced highly purified cardiomyocytes (more than 90%) by optimization of the culture condition [60]. Cao et al. reported that ascorbic acid robustly enhanced cardiomyogenesis of all 11 lines so that differences were smaller [61]. Ascorbic acid functioned to proliferate cardiomyocyte progenitors. Ribosomal S6 kinase [62] and mitogen-activated protein kinase (MAPK) activities [63] affected cardiomyogenesis. Some small molecules had been known to have effect on cardiomyogenesis. Previously investigated effects of 36 small molecules using ES cells were summarized [64]. In addition to that, recently, small molecule, dorsomorphin, an inhibitor of BMP signaling [65], and XAV929, an inhibitor of Wnt/*β* signaling [45, 66], promoted cardiomyogenesis. Cyclosporin-A [67], sulfonyl hydrazone-1 [68], and even a simple dissociation of EBs [69] enhanced cardiomyogenesis. These molecules will help to accelerate cardiomyogenesis. However, a more concise profiling of molecular signatures is necessary to evaluate maturity and function.

Recently, a unique method to purify cardiomyocytes using the high number of mitochondria within cardiomyocytes was reported [70, 71]. In this method, genetic engineering is not required, and damage to cells should be decreased. On the other hand, another method was established using the signal-regulatory protein alpha (SIRPA), which can select immature cardiomyocytes which have fewer mitochondria [72].

## 4. Future Model of Heart Disease Composed of the iPS-Cells-Derived Cardiomyocytes

Very recently, in a genomic mutation heterozygous for polysystic kidney disease 1 (Pkd1), the deletion is restored by spontaneous mitotic recombination [73]. Indeed, the frequency of genetic repair events by spontaneous mitotic recombination in pluripotent stem cells is higher than that in somatic cells [74]. Interestingly, from the RT-PCR data from Cheng et al., not only wild-type iPS cells but also −/− iPS were detected [73]. These results are also important to heart diseases, especially for dominant mutation. Comparison of these (+/+ and −/−) cells can be perfect because there is no genetic background difference, since they are derived from the same person.

Currently, several multielectrode array systems for *in vitro* extracellular electrophysiology are available for QT prolongation screening with iPS cell-derived myocytes. In order to screen the function of mutated channels located on subcellular organelles such as the RYR2, fluctuations in intracellular Ca^2+^ concentrations should be measured. Development of a user-friendly detection system for stimulation and recording of such channels in patient cardiomyocytes is expected [75], as well as sensor techniques and bioanalytical approaches for cardiotoxicity testing [76].

Because tissues are three dimensional, 3D *in vitro* models can be made using scaffolds [77] or cell sheets [78, 79] in the near future. The process of tissue formation can be observed and compared with normal tissue formation. For this purpose, not only cardiomyocytes, but also other cardiac cells should be developed. Hearts contain a vascular system, which is difficult to constitute using a 2D model; however, it may be possible using a 3D model [80].

Gene expression levels [81, 82] and protein profiles [83] can be analyzed similarly to other cell culture systems. Recent progresses in the investigation of micro-RNA have provided information on the process to disease. Micro-RNA can be biomarkers for cardiovascular diseases [84] and have gained attention as regulators for cardiac injury and protection [85]. Cardiac differentiation by BMP from cardiac progenitors was mediated by micro-RNA [86]. In fact, micro-RNA is associated with cell fate decision [87]. In cardiomyocyte differentiation, miR-1 and miR-133 are upregulated, and miR-499 promotes cardiomyogenesis [88]. Thus, the state of the disease can be more precisely assessed by micro-RNA expression. Networks of mRNA and micro-RNA to determine human cardiomyocytes differentiation were investigated [89], and such attempts should be required, and analytical development is also required to fit this. Not only gene expression, but also global methylation analysis of CpG islands and the identification of non-CpG islands by next generation sequences is also useful. Other epigenetic approaches should make progress in this field [90]. Because some differences were reported between iPS-cell-derived cardiomyocytes and tissue-derived cardiomyocytes, where iPS cell-derived cardiomyocytes were more immature than tissue derived cardiomyocytes, further studies should be performed to evaluate their quality.

## 5. Conclusion

Using iPS cells for *in vitro* heart disease models is a promising method for evaluating drug effects. Many disease models should be constructed. However, further studies are necessary to evaluate cardiomyocytes in terms of heterogeneity using molecular biological analyses derived from the patient's tissues.

## Figures and Tables

**Table 1:** HRS/EHRA Expert Consensus Statement on Genetic Testing (Heart Rhythm 2011; 8 : 1308–1339).

Cardiac Channelopathy /Cardiomyopathy	Diagnostic implications of genetic testing	Class I	Class IIa	Class IIb	Class III	Testing genes	Common disease genes	
		“is recommended”	“can be useful”	“may be considered”	“is not recommended”		Genes	% of disease
Long QT syndrome (LQTS)	Patient in whom a cardiologist has established a strong clinical index of suspicion for LQTS	○				Comprehensive or LQT1-3		
	Asymptomatic patient with QT prolongation in the absence of other clinical conditions that might prolong the QT interval on serial 12-lead ECGs defined as QTc >480 ms (prepuberty) or >500 ms (adults)	○					*KCNQ1 *(LQT1) *KCNH2 *(LQT2) *SCN5A *(LQT3) LQT4-13	*30–35%* *25–40%* *5–10%* *>5%*
	Asymptomatic patient with otherwise idiopathic QTc values >460 ms (prepuberty) or >480 ms (adults) on serial 12-lead ECGs			○		Mutation-specific
	Family members and other appropriate relatives subsequently following the identification of the LQTS-causative mutation in an index case	○				

Catecholaminergic polymorphic ventricular tachycardia (CPVT)	Patient in whom a cardiologist has established a clinical index of suspicion for CPVT	○				Comprehensive or CPVT1 and CPVT2	*RYR2 *(CPVT1)	60%
	Family members and appropriate relatives following identification of the CPVT-causative mutation in an index case	○				Mutation-specific	*CASQ2 *(CPVT2)	3–5%

Brugada syndrome (BrS)	Family members and appropriate relatives following identification of the BrS-causative mutation in an index case	○				Mutation-specific	*SCN5A* (BrS1)	20–30%
	Patient in whom a cardiologist has established a clinical index of suspicion for BrS		○			Comprehensive or *SCN5A *		
	The setting of an isolated type2 or type3 Brugada ECG pattern				○	—		

Progressive cardiac conduction disorders (CCD)	Family members and appropriate relatives following the identification of the CCD-causative mutation in an index case	○				Mutation-specific	*SCN5A*	5%
	Patients with either isolated CCD or CCD with concomitant congenital heart disease, especially when there is documentation of a positive family history of CCD			○		*SCN5A and TRPM4*		

Short QT syndrome (SQTS)	Family members and appropriate relatives following the identification of the SQTS-causative mutation in an index case	○				Mutation-specific		
							*KCNH2* (SQT1) *KCNQ1* (SQT2) *KCNJ2* (SQT3)	>5% >5% >5%
	Patient in whom a cardiologist has established a strong clinical index of suspicion for SQTS based on examination of the patient's clinical history, family history, and electrocardiographic phenotype			○		Comprehensive or SQT1-3		

Atrial fibrillation (AF)	Genetic testing is not indicated for atrial fibrillation at this time.				○	—	None of the known	
							disease-associated	
							genes has been	
							shown to account	
							for >5% of this disease.	

Hypertrophic cardiomyopathy (HCM)	Patient in whom a cardiologist has established a clinical diagnosis of HCM	○				Comprehensive or targeted (*MYBPC3, MYH7, TNNI3, TNNT2*, and *TPM1*)	*MYBPC3* *MYH7* *TNNT2* *TNNI3* *TPM1*	20–45% 15–20% 1–7% 1–7% >5%
	Family members and appropriate relatives following identification of the HCM-causative mutation in an index case	○				Mutation-specific		

Arrhythmogenic cardiomyopathy (ACM)/Arrhythmogenic right ventricular cardiomyopathy (ARVC)	Family members and appropriate relatives following identification of the ACM/ARVC-causative mutation in an index case	○				Mutation-specific	*PKP2* *DSG2* *DSP* *DSC2* *TMEM43*	25–40% 5–10% 2–12% 2–7% >5%
	Patients satisfying task force diagnostic criteria for ACM/ARVC		○			Comprehensive or targeted (*DSC2, DSG2, DSP, JUP, PKP2*, and *TMEM43*)		
	Patients with possible ACM/ARVC (1 major or 2 minor criteria) according to the 2010 task force criteria (European Heart Journal)			○				
	Patients with only a single minor criterion according to the 2010 task force criteria				○	—		

Dilated cardiomyopathy (DCM)	Patients with DCM and significant cardiac conduction disease (i.e., first-, second-, or third-degree heart block) and/or a family history of premature unexpected sudden death	○						
	Family members and appropriate relatives following the identification of a DCM-causative mutation in the index case	○				Comprehensive or targeted (*LMNA* and *SCN5A*)	*LMNA* *SCN5A*	>5% >5%
	Patients with familial DCM to confirm the diagnosis, to recognize those who are at highest risk of arrhythmia and syndromic		○			Mutation-specific		
	features, to facilitate cascade screening within the family, and to help with family planning					Mutation-specific		

Left ventricular noncompaction (LVNC)	Family members and appropriate relatives following the identification of an LVNC-causative mutation in the index case	○				Mutation-specific *LBD3*, and so forth	*LBD3*	*∼*5%
	Patients in whom a cardiologist has established a clinical diagnosis of LVNC		○					

Restrictive cardiomyopathy (RCM)	Family members and appropriate relatives following the identification of an RCM—causative mutation in the index case	○				Mutation-specific	**β*-MHC*	*∼*5%
	Patients in whom a cardiologist has established a clinical index of suspicion for RCM			○		*MYH7, TNNI3*, *TNNT2 *	*TNNI3*	*∼*5%

Out-of-hospital cardiac arrest survivors	The survivor of an Unexplained Out-of-Hospital Cardiac Arrest	○				Appropriate genes following diagnosis of the survivor —	*RYR2* *KCNQ1* *KCNH2*	10–15% 5–10% *∼*5%
	Routine genetic testing, in the absence of a clinical index of suspicion for a specific cardiomyopathy or channelopathy				○			

Postmortem genetic testing in sudden death cases (SUD/SIDS)	For all SUDS and SIDS cases, collection of a tissue sample	○						
	In the setting of autopsy negative SUDS			○		comprehensive or targeted (*RYR2, KCNQ1, KCNH2, and SCN5A*) Mutation-specific	*RYR2* *KCNQ1* *KCNH2* *SCN5A*	*SCN5A*: 3–5%
	Family members and other appropriate relatives following identification of a SUDS-causative mutation in the decedent	○						

HRS: the Heart Rhythm Society, EHRA: European Heart Rhythm Association. We summarized their tables with permission.
